# Femtosecond manipulation of spins, charges, and ions in nanostructures, thin films, and surfaces

**DOI:** 10.1063/1.4995541

**Published:** 2017-12-19

**Authors:** F. Carbone, M. Hengsberger, L. Castiglioni, J. Osterwalder

**Affiliations:** 1 Ecole Polytechnique Fédérale de Lausanne, Institute of Physics, Laboratory for Ultrafast Microscopy and Electron Scattering (LUMES), EPFL Campus, Lausanne, Dorigny CH-1015, Switzerland; 2 Department of Physics, University of Zurich, CH-8057 Zurich, Switzerland

## Abstract

Modern ultrafast techniques provide new insights into the dynamics of ions, charges, and spins in photoexcited nanostructures. In this review, we describe the use of time-resolved electron-based methods to address specific questions such as the ordering properties of self-assembled nanoparticles supracrystals, the interplay between electronic and structural dynamics in surfaces and adsorbate layers, the light-induced control of collective electronic modes in nanowires and thin films, and the real-space/real-time evolution of the skyrmion lattice in topological magnets.

## I. INTRODUCTION AND MOTIVATION

### A. Condensed matter and strongly correlated systems

In novel low-dimensional materials, topological protection and/or electronic correlations lead to exotic charge, spin, or orbitally ordered ground states having new functionalities such as multiferroelectricity, high-temperature superconductivity, skyrmion magnetism, and Weyl semimetallicity, to name a few.^1^ Such ordered spatial textures exhibit atomic to few-nm characteristic lengths, and their dynamical behavior has fs to ps characteristic times. Furthermore, it is increasingly evident that the ground state of several strongly correlated materials is spatially inhomogeneous.^2,3^ The current grand-challenge in experimental condensed matter physics is to move from spectroscopy and reciprocal-space microscopy to real-space/real-time techniques providing direct access to such phenomenology.^4^

### B. Soft matter and nanostructures

New functionalities for drug delivery, plasmonic devices, energy harvesting, and sensing are also emerging in new materials assembled out of nano-particles of different shapes and sizes. It is a fascinating subject of research at the verge between condensed and soft matter physics that can be tackled by modern ultrafast techniques.^5^

Supracrystals composed of metallic nanoparticles coated with specific ligand molecules can be designed and fabricated via self-assembly methods. The ligands attached to the nanoparticles (NP) can be used to provide specific functionalities, thanks to their interaction with the environment.^6^ The applicability of functionalized gold nanoparticles in catalysis, biosensing, drug delivery, plasmonics, or solar energy harvesting depends on the ordering properties of the organic ligands shell coating them. For this reason, an intense debate started recently in the literature on whether or not self-organization phenomena among the ligands can be obtained and observed. A conclusive answer to these questions can be provided solely by a technique capable of obtaining atomically resolved information on the structural properties of both the NPs and the ligands at the same time. We will show that fs small angle electron diffraction combined with the analysis of angular cross-correlation functions (CCF, see below) allows for tracking the concerted motion of ions and ligands during the dynamical evolution of self-assembled supracrystals, providing the structural characterization needed to model the function of such systems.^7,8^ In a liquid environment instead, the limited penetration depth of electrons prevents their utilization as a probe. X-ray techniques on the contrary can access such mixed liquid-solid phases and provide complementary information.^9,10^

### C. Surfaces

The surface is the interface between any material and its environment. Beside the fact that atomic arrangement and electronic properties often change between bulk and surface, the chemical activity is determined by surface properties. The latter is important for heterogeneous catalysis or inertness against corrosion, for instance. Moreover, with the advent and big success of dye-sensitized surfaces for solar cells^11^ and for photo-catalysts,^12^ the optimization of molecular adsorption and charge transfer between substrate surface and molecule became the most prominent route to control the chemical activity of surfaces. The importance was highlighted by awarding the Nobel Prize in chemistry in 2007 to Ertl (FHI Berlin) for his “studies of photochemical processes on surfaces.”

Eventually, decisive properties like charge transfer, molecular dipole, and sticking coefficient for molecular adsorption are solely determined by the local coordination of the adsorption sites, and thereby, by the structural arrangement of molecule and surface atoms. Due to the high surface sensitivity of electron-based methods, (photo-) electron diffraction in general is one of the most important tools to study adsorption geometries and surface reconstructions.

### D. Motivation

Modern ultrafast technology promises to address these fundamental challenges. The ability to record fs and sub-nm resolved movies of atoms, spins, and charges will provide a hitherto unsurpassed degree of insight into such phenomena. As a matter of fact, literally billions of dollars are being spent worldwide in large-scale installations like free-electron lasers to ensure that such goals are reached and ultrafast science has never been as popular as nowadays.

Furthermore, all-optical control of these states of matter would pave the way to exploit these novel functionalities for applications.

In this review, we will discuss the recent results obtained via dynamical imaging methods with a particular focus on electron-based techniques.

First, we will provide a concise introduction on the experimental methods available within MUST to perform time-resolved imaging of spin, charges, and atoms. These will include dynamical transmission electron microscopy (TEM),^13^ time-resolved electron diffraction,^14^ and photoelectron diffraction.^15^

We will then discuss specific examples: (i) Dynamical imaging of charges: in this example, we will show that the collective charge excitations in nanostructures and 2D thin films can be controlled at the fs/nm scale via Photon-Induced Near field Electron Microscopy (PINEM).^16^ (ii) Dynamical imaging of spins: in this case, we will demonstrate the importance of obtaining time-resolved (ms) and spatially resolved information on spin motions in topological magnets.^17^ (iii) Dynamical imaging of atoms: fs-resolved movies of the photoinduced dynamics of a self-assembled gold nanoparticles supracrystal were obtained by the newly developed fs-small angle electron diffraction.^7^ These data revealed the possibility to control the order/disorder transition in glassy aggregates. (iv) The structural response of the Bi111 surface: Bismuth is a well-known model system for investigating the interplay between collective modes and the electronic structure. Absorption of infrared light leads to a large displacement of the electronic charge. Due to the new charge order, the atomic equilibrium positions change. The consequent coherent structural distortions can be tracked by ultrafast photoelectron spectroscopy. (v) Conformational changes in molecules: in tetra-tert-butyl-azobenzene (TBA)/Au(111), switching between different structural configurations can be obtained via light pulses. The fraction of switched molecules and the switching rates can be determined using X-ray photoelectron diffraction, obtaining direct structural and time-resolved information on the photoexcited process.

A discussion on the perspectives of these results will follow, with a particular attention to their connection with similar studies performed via complementary X-ray scattering methods.

## II. METHODOLOGIES

The technique of reference in time-resolved experiments is pump-probe. Conceptually, the need for acquisition times far exceeding the femtosecond time-scale causes the necessity of using a clocking pulse (pump) capable of initiating a reversible process at well-defined times. The status of the given process at a time t_1_ after its initiation (t_0_) can then be frozen by recording a sequence of probe events delayed from the pump by t_1_–t_0_. Such a stroboscopic experiment is the most common route to time-resolved measurements. Since the inception of ultrafast lasers, infrared-visible light has become the natural tool for ultrafast spectroscopy. In recent years, significant technological advances in the generation of ultrashort electron pulses enabled a variety of other techniques to complement the above-mentioned light-based spectroscopies. Electrons offer a unique cross section for interaction with matter (one million times higher than X-rays for example) and a very short associated wavelength (at comparable energy). These two characteristics make them particularly suited for the investigation of very thin films, low dimensional materials, and nanostructures, as well as for microscopy application all the way down to atomic spatial resolution.^4^

**FIG. 1.**
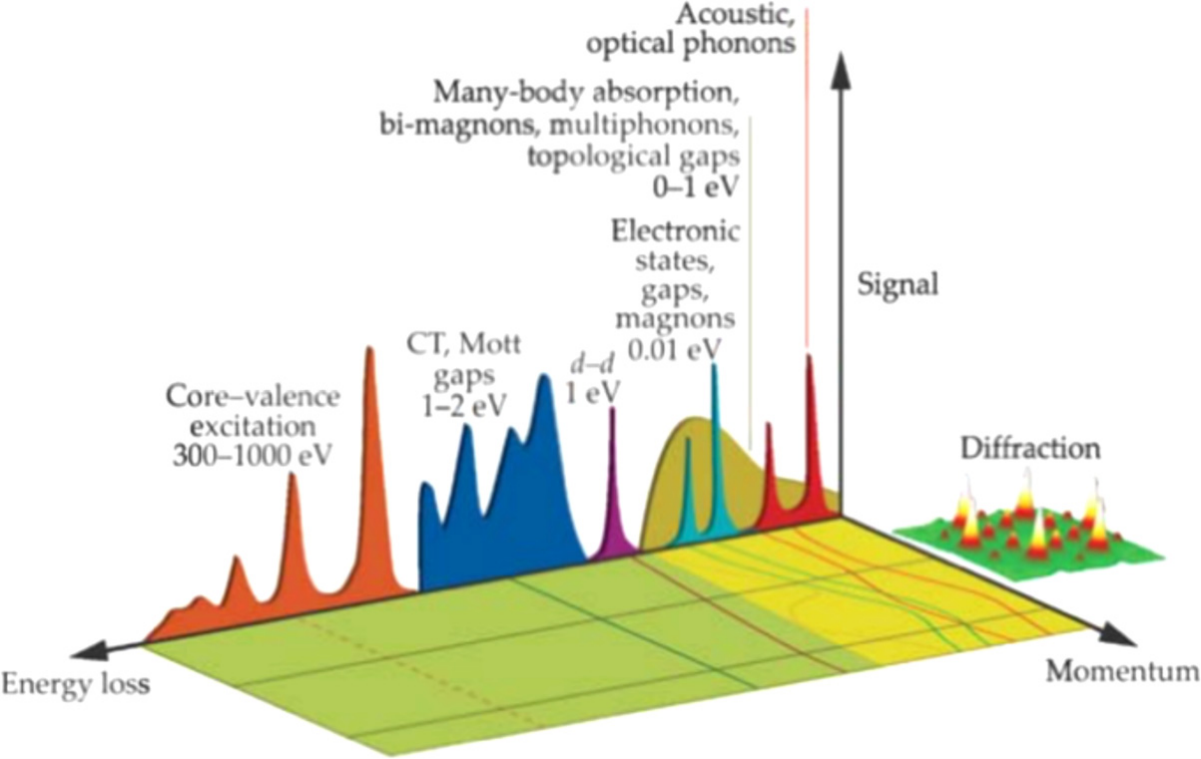
Taxonomy of the electronic/structural and magnetic excitations in solids (this image is reproduced with permission from Y. Zhu and H. Durr, Phys. Today **68**(4), 32 (2015). Copyright 2015 AIP Publishing LLC).^72^

In Fig. 1, we classified the relevant electronic and structural excitations of interest in materials on an energy/momentum landscape. Conventional ultrafast optical tools are mostly restricted to the exploration of the energy axis of this diagram, with some momentum resolution being allowed for example by diffraction or Raman-based techniques such as resonant inelastic X-ray Scattering (RIXS, only recently demonstrated with fs-resolution at a free electron laser^18,19^) or impulsive stimulated Raman scattering (ISRS).^20,21^ Energy and momentum resolution can be readily obtained instead via time and angle resolved photoelectron spectroscopy (TR-ARPES).^22^

Electron-based ultrafast techniques offer a valid alternative as they can cover a vast portion of this landscape with a combination of diffraction and spectroscopy experiments. Thanks to an increasing energy resolution in modern machines, many-body structural and electronic excitations close to the Fermi level are becoming accessible. Beyond these, interband transitions, Mott gaps, charge transfers, core-levels, and dispersive excitations in the few hundreds of meV to eV range, such as plasmons, are easily accessible by electron energy loss spectroscopy.^23^ Furthermore, when implemented in a transmission electron microscope (TEM), spectroscopy can be combined with atomic-resolution imaging, offering a unique perspective for the investigation of inhomogeneous states or exotic charge and spin spatial textures. Also, Lorentz TEM provides sensitivity to very weak magnetic fields with spatial resolution down to 1 nm.^24^

### A. Time-resolved transmission electron microscopy

**FIG. 2.**
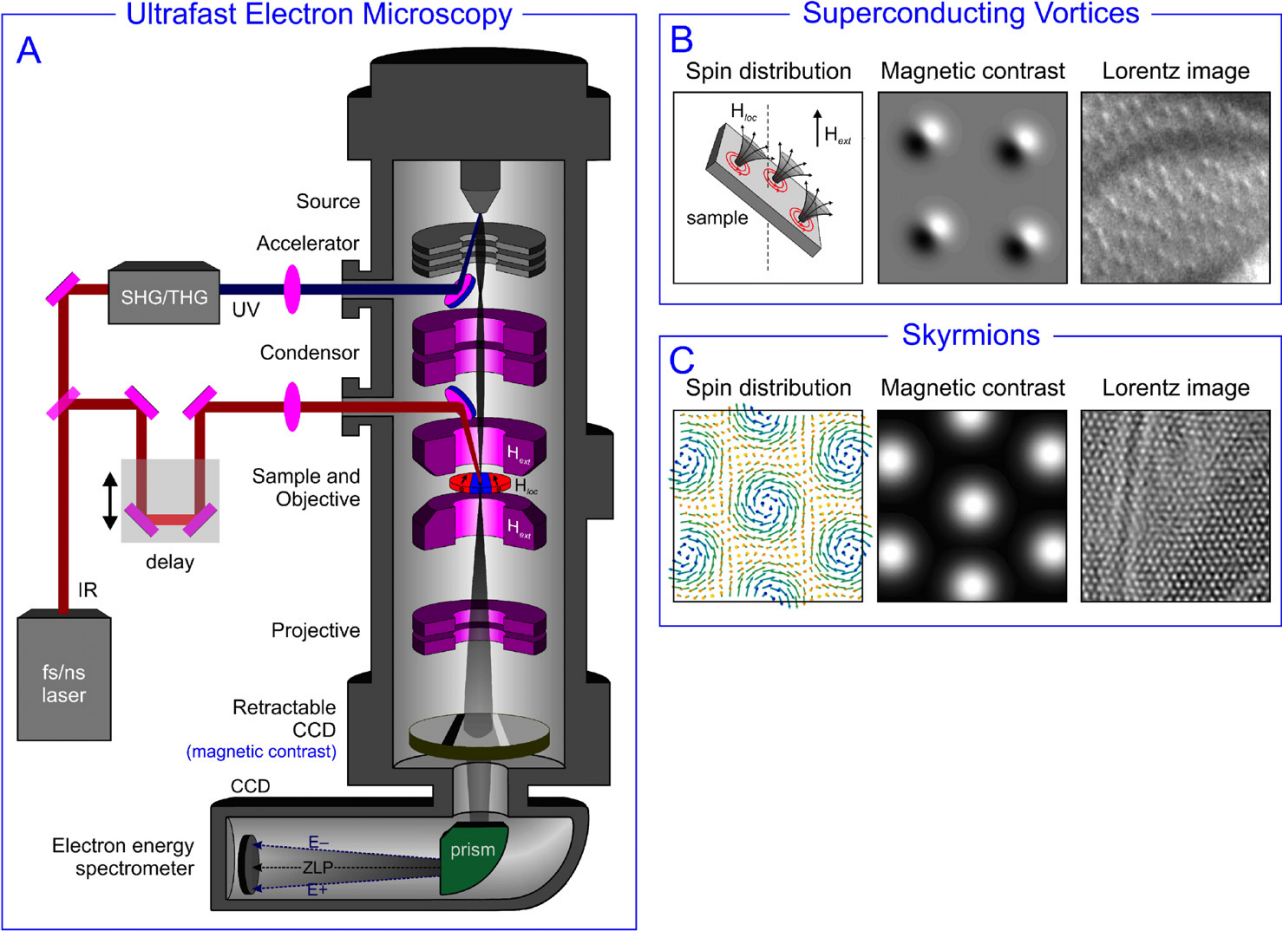
Ultrafast transmission electron microscopy. (a) Schematics of a time-resolved TEM. (b) and (c) Principle and example of Lorentz imaging of a superconducting vortex lattice and a skyrmion lattice, respectively.

Schematics of this technique is depicted in Fig. 2(a). In brief, a 100 kHz–1 MHz train of 50-fs light pulses is split to generate two beams. One is frequency tripled to deliver ultraviolet pulses which photoemit electrons from a cathode in a modified TEM. The other 800-nm pulses (or different λ if converted in a nonlinear device) pass through an optical delay line and are focused on the sample. The Electron Energy Loss Spectrometer (EELS) analyzes the energy of the electrons scattered from the specimen while preserving the information about their trajectories for energy-filtered imaging purposes. The electrons are eventually detected by a Charge-Coupled Device (CCD). In such a TEM, ultrafast imaging,^25^ diffraction,^26^ and spectroscopy^27^ can be achieved. Furthermore, combinations of these are also possible,^28^ providing unique information on plasma resonances (PRs) and the momentum-resolved electronic structure of materials. In a TEM, magnetic contrast can be obtained by detecting electrons scattered by a magnetic field at a very small angle, 10^−6 ^rad; such a technique is referred to as Lorentz microscopy.^29^ To do this, one needs to record electrons that transmit and propagate away from the specimen over a long distance by defocusing images (Fresnel mode). In Figs. 2(b) and 2(c), an example of Lorentz images of superconducting vortices and magnetic skyrmions is shown.

### B. Time-resolved electron diffraction

**FIG. 3.**
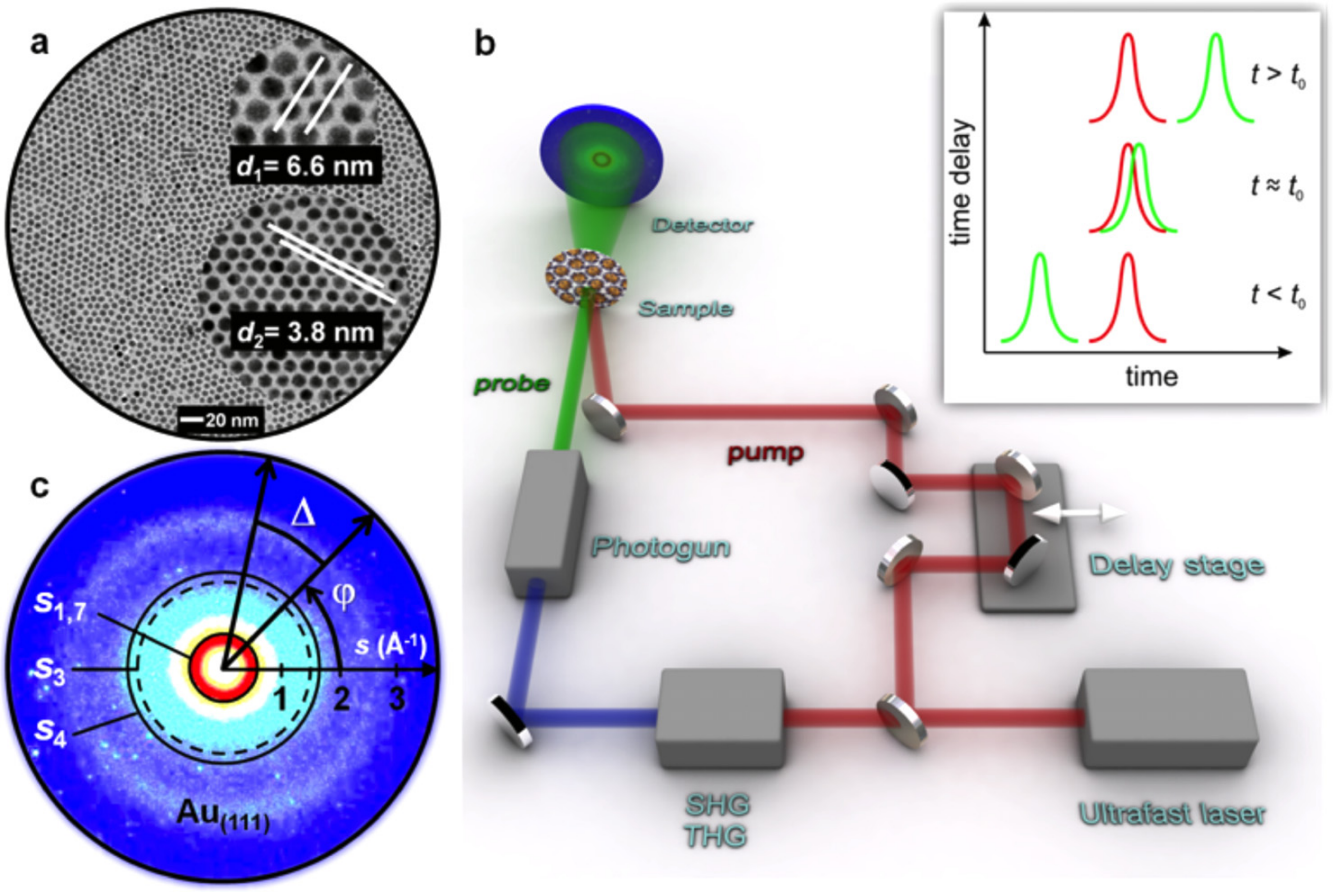
Ultrafast small-angle electron diffraction from a NP supracrystal. (a) Self-assembled gold NP supracrystal. (b) Schematics of the experimental apparatus. (c) Amorphous diffraction pattern from the supracrystal in (a); in this image, the angels and distances necessary for the computation of the cross-correlation function (CCF) are shown, see text. Reproduced with permission from Mancini *et al.*, Nano Lett. **16**(4), 2705–2713 (2016). Copyright 2016 American Chemical Society.

30 kV electrons can be generated by sending a frequency-tripled ultrafast 800 nm laser, 50 fs pulse duration, 20 kHz repetition rate on a back-illuminated photocathode. High-brightness, ultrashort (<300 fs) bunches were obtained by radiofrequency compression, compensating for space charge broadening, and were focused with a set of magnetic lenses to a ≈160 *μ*m spotsize onto a sample. Electron diffraction patterns can be obtained in our setup both in reflection and transmission geometry, allowing for the investigation of surfaces and thin films. The impulsive photoexcitation of each sample is obtained with 1.5 eV light pulses with ≈250 *μ*m spotsize at the sample plane. Different energies can be used by converting the fundamental frequency of the laser output via the use of non-linear optical devices.^14^ In reflection geometry, the group velocity mismatch between the electrons and the photon pulses is compensated by tilting the optical wavefront of the pulses, see Refs. 30 and 31. Schematics of this setup is represented in Fig. 3(b).

### C. Time-resolved photoelectron diffraction

Photoelectron diffraction relies on the coherent scattering of photoelectrons at atoms in vicinity of the emitter atom. Since the path length of the photoelectron is limited by the inelastic mean-free path, the interference pattern represents only the local environment of the emitter (see, e.g., Ref. 15 and references therein). Compared to photoelectron diffraction, conventional electron diffraction patterns are images of reciprocal space and are sensitive to long-range order. Since the final image in conventional electron diffraction is a superposition of many diffraction patterns of single electrons, the range probed in real space essentially depends on the so-called coherence properties of the electron beam, i.e., its monochromaticity and beam divergence.^32^

**FIG. 4.**
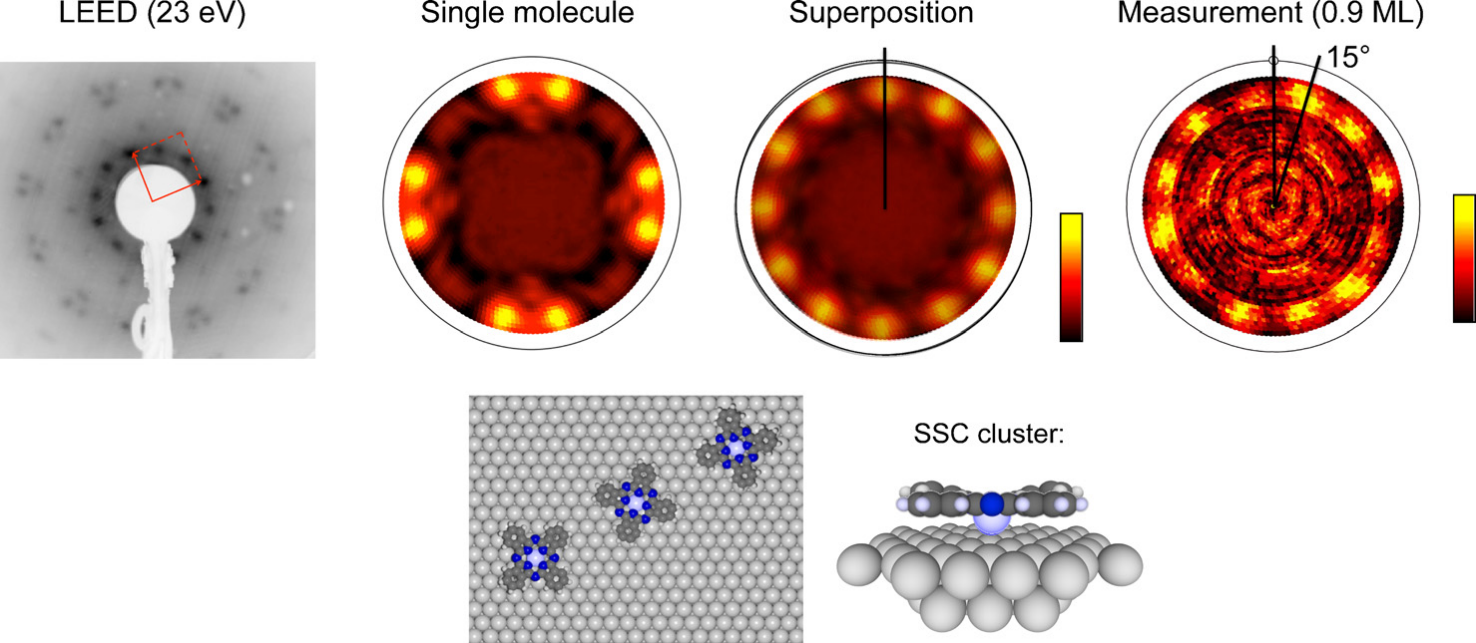
Photoelectron diffraction data from Sn-phthalocyanine (SnPc) adsorbed on an Ag(111) surface. The LEED picture on the left-hand side shows the appearance of three rotational domains, each with a set of unit cell vectors (as shown by the red arrows). The single scattering cluster (SSC) simulations are displayed for the case of a single SnPc molecule with four-fold symmetry, and the pattern obtained by a sum over such patterns for each domain on the three-fold surface. The 12 spots at the outer rim can be compared to the measurement shown on the right-hand side and allow to determine the absolute orientation with respect to the substrate. Reproduced with permission from Greif *et al.*, Phys. Rev. B **87**, 085429 (2013). Copyright 2013 American Physical Society.

In contrast to conventional diffraction methods, photoelectron diffraction is not sensitive to long-range order. Nonetheless, a typical pattern represents the integration over many (up to 10^13^–10^14^) emitter atoms. Therefore, a pattern with good contrast requires the number of differently oriented domains to be as small as possible. Moreover, in the case of molecules this means that contrary to conventional electron diffraction, photoelectron diffraction is sensitive to the orientation of molecules rather than to the arrangement of an ensemble of molecules, as demonstrated in Fig. 4. Dephasing of a coherently excited ensemble of molecules can be measured as loss of signal contrast with time due to averaging over many different molecular coordinations.^33^

## III. DYNAMICAL IMAGING OF CHARGES, PHOTON-INDUCED NEAR FIELD ELECTRON MICROSCOPY

The observation of surface plasmon polaritons (SPP) with nm-fs combined resolution has been achieved by few optical techniques such as dual-color coherent anti-Stokes Raman scattering (CARS)^34^ and scanning near-field optical microscopy (NSOM).^35^ Also, time-resolved nonlinear photoemission electron microscopy (TR-PEEM)^36^ has been shown to achieve a temporal resolution down to few-fs. Both NSOM and TR-PEEM provide some sensitivity to SPPs confined at interfaces between different layers.^37,38^ However, since TR-PEEM relies on photoelectrons emitted from a surface and scanning-tip probes require rastering a sample in proximity to its surface, they are both limited in sensing electromagnetic fields leaking outside a surface. Furthermore, in these experiments, the relation between the probed field and the photonic local density of states is not direct. This can be overcome in PEEM at normal incidence.^39^ These restrictions prevent the study of heterostructures and multilayers. EELS instead directly probes the density of state of the whole sample via electrons propagating along the z-direction. In conventional EELS experiments, all the plasma resonances of nanostructures are excited by the transient electric field of the electron beam crossing the sample.^40^

When looking at low energy plasmons, EELS experiments are typically limited by the monochromaticity of the electron beam. In machines equipped with cold field emission guns and monochromators, using scanning methods, the energy resolution can be pushed below 100 meV.^23^ However, this approach cannot be applied to a time-resolved instrument because of the limited beam brightness available in this modality.

**FIG. 5.**
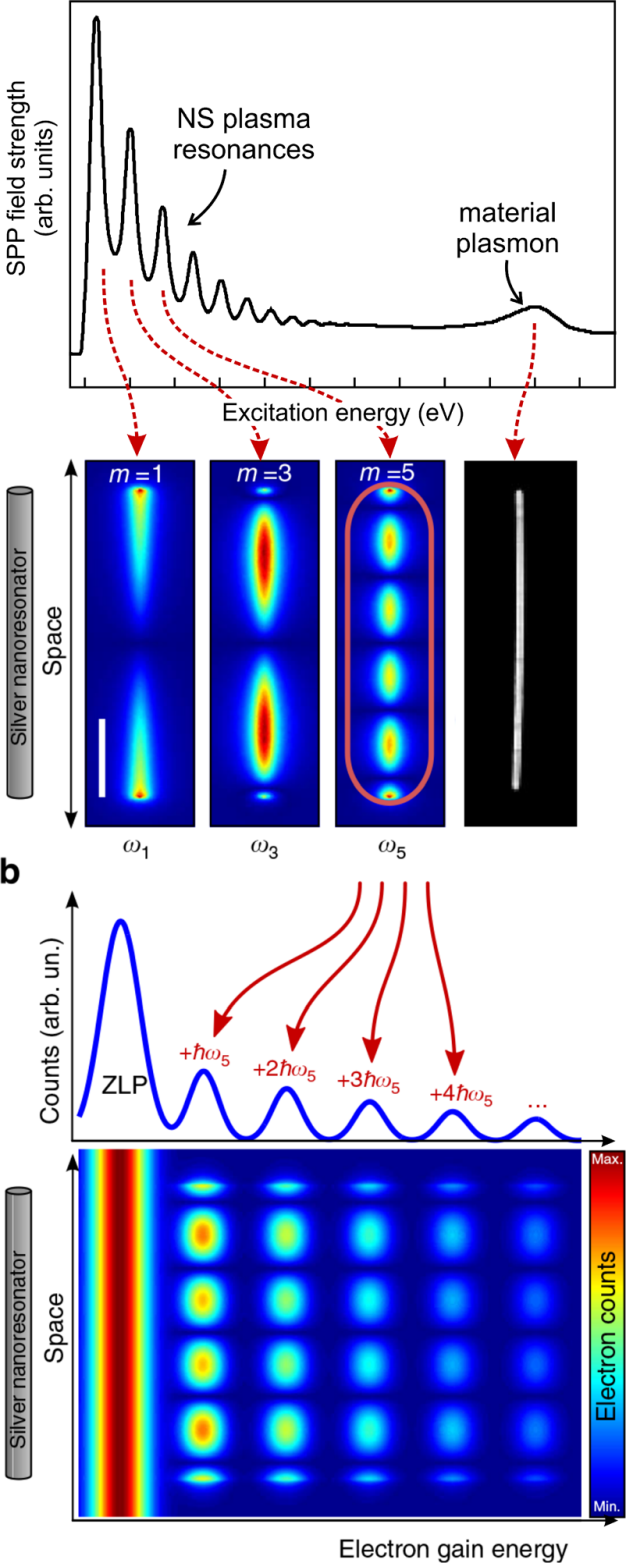
Photon induced near field electron microscopy (PINEM). An EELS spectrum of a silver nanowire showing the plasma resonance (PR) visualized via spectral imaging in real space. (b) PINEM principle: laser-excitation of one PR and consequent space-energy electron scattering distribution.

**FIG. 6.**
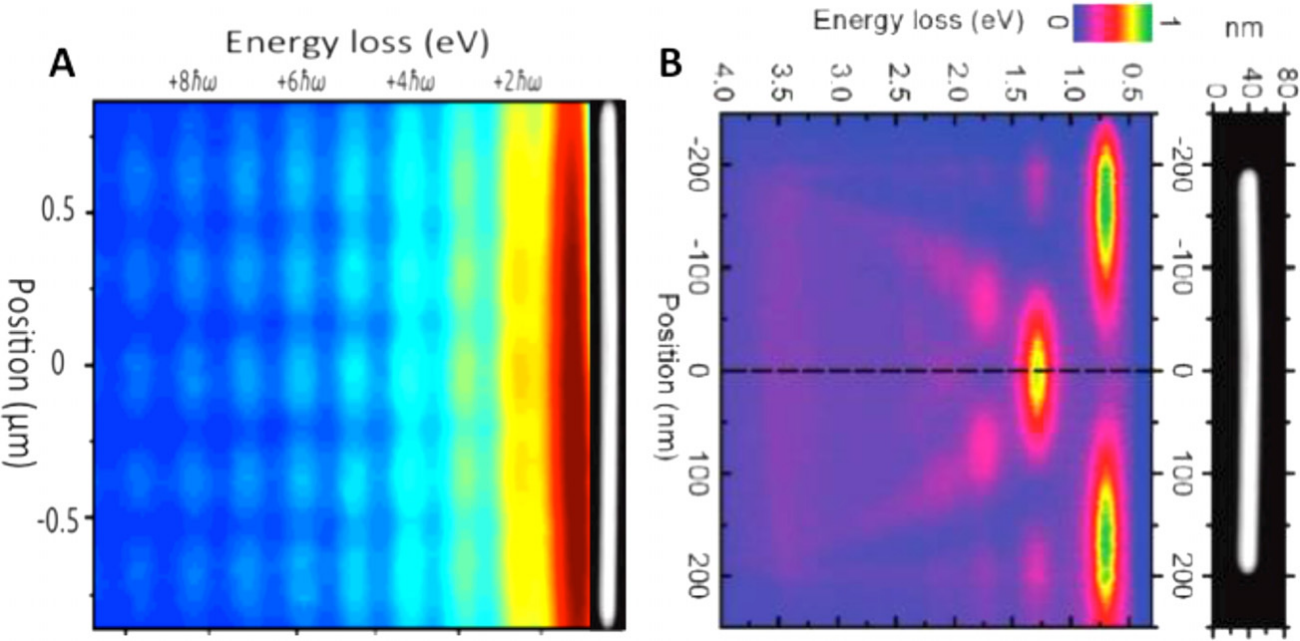
Comparison between PINEM and Spectral imaging. (a) Space-energy map of a PR as visualized by PINEM. (b) Space-energy map of conventional spectral imaging. Reproduced with permission from Rossouw *et al.*, Nano Lett. **11**, 1499 (2011). Copyright 2011 American Chemical Society.

A new powerful method to characterize plasma resonances based on ultrafast electron microscopy is photon induced near field electron microscopy.^16^ A plasma resonance is excited by a laser pulse, see Fig. 5(a). For a metallic nanowire, SPPs trapped between its edges yield different plasma resonances. If the light excitation is monochromatic, the resulting EELS spectrum gets broadened by the multiple energy exchanges with the low-energy PR selected by the laser energy [see orange trace in Fig. 5(b)]. This gives rise to several peaks in the energy loss/gain spectrum, each representing the exchange of an integer number of the energy of the laser-driven mode [see Fig. 5(b)]. By imaging the inelastic electrons in energy filtered electron microscopy (EFTEM), the spatial profile of the plasma resonance itself can be obtained.^28^ To highlight the difference between the plasma resonances energy and the quantization of each plasmon mode itself in the photo-excited and conventional EELS spectra, we developed a methodology in our laboratory that allows for a simultaneous characterization of the spatial profile of an SPP and its spectrum.^28^ In such an energy-space map, the spatial profile of the plasma resonance and its order [i.e., the number of nodes in the Fabry-Perot (FP resonator)] is seen not to vary as a function of energy. Nevertheless, the spectral profile of the energy exchange between the SPP and the imaging electron shows multiple peaks that are instead a signature of the quantized nature of the plasmonic field. In contrast, conventional spectral imaging shows that higher order plasma resonances (i.e., having more nodes) are found at higher energies.^40^ This difference is depicted in Figs. 6(a) and 6(b) where energy-space mapping of plasma resonances is carried out in PINEM and spectral imaging.

These experiments provided a visual microscopic description of the PINEM effect.

Furthermore, the extra degree of freedom offered by the possibility to excite plasmons with light pulses enables the control of the plasma resonances shape by tuning light polarization with respect to the geometry of different nanofabricated surfaces.^28,41^ In Ref. 42, the propagation speed of plasmons in a silver thin film was also obtained via this method, providing a powerful tool for the characterization of the plasmonic response of multilayered materials.^41^ In fact, the strongly reduced propagation speed of plasmons in this report was attributed to the confinement imposed by the very thin film. The same method will apply to the investigation of highly confined plasmonic fields in low-dimensional materials.

## IV. DYNAMICAL IMAGING OF SPINS, TIME-RESOLVED LORENTZ ELECTRON MICROSCOPY

The characterization and control of topological spin patterns all the way down to the fs-nm scale has recently driven a lot of attention for both fundamental reasons and possible applications. In this section, we shall provide an example of the advantage of the real-time/real-space characterization of the dynamics of the skyrmion lattice, which is a periodic arrangement of whirling distribution of spins, called skyrmions.^43^

Imaging and spectroscopic studies of skyrmions have shown their shape and microscopic origin in various materials;^43–46^ however, the critical information on their motions, evolution across a phase transition, and response to an impulsive excitation is limited by the difficulty in obtaining time-domain images of nm-sized magnetic textures.

**FIG. 7.**
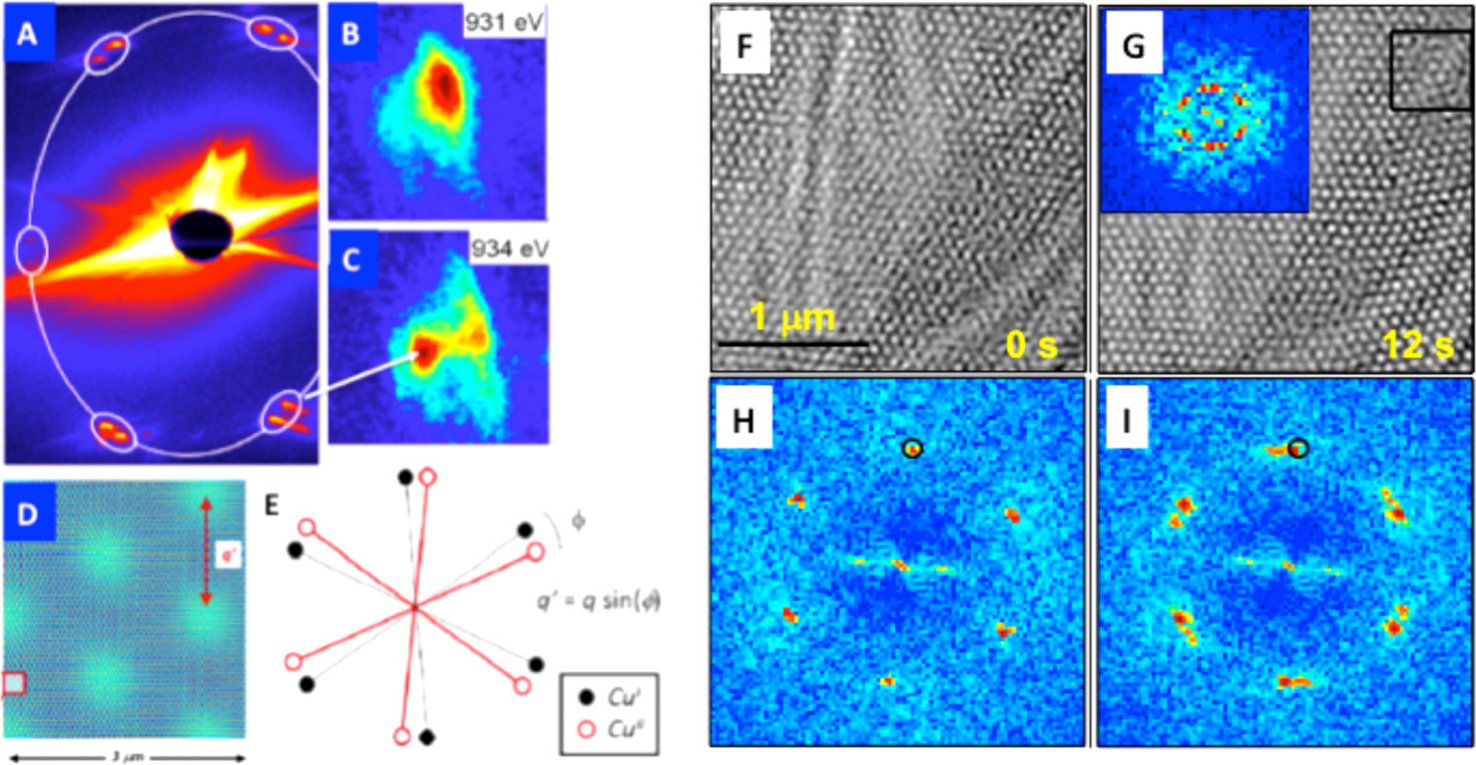
Reciprocal-space vs real-space dynamical imaging of the skyrmion lattice. (a) Resonant X-ray diffraction pattern from Cu_2_OSeO_3_. (b) and (c) Splitting of the magnetic satellites. (d) Simulation of a Moiré pattern resulting from the skyrmions distribution in (e). (e) Misoriented skyrmion lattice. (f)–(i) Lorentz TEM images at different times. Panels (a)–(e) are adapted from Ref. 49 and panels (f)–(i) are adapted from Ref. 17.

In B20 chiral materials such as Cu_2_OSeO_3_, the anysotropic exchange interaction and the Dzyaloshinkii-Moriya interaction^47^ lead to a helical distribution of spins that can result in the formation of a skyrmion lattice.^48^ The individual magnetic moment of the unit cell originates from the net sum of two sublattices of spins present in two non-equivalent sites of the crystal.^44^ Recently, it has been argued that the magnetic diffraction peak associated with the skyrmion lattice observed by resonant X-ray diffraction, see Fig. 7(a), could be split into two satellites by applying a magnetic field, in Figs. 7(b) and 7(c);^49^ each of these satellites resonated at a different energy within the Cu L-edge absorption threshold.

This behavior was ascribed to the existence of two misaligned sub-lattices of skyrmions, giving rise to a Moiré pattern in real space, see Figs. 7(d) and 7(e).

To test for this hypothesis, we performed camera-rate resolved cryo-Lorentz transmission electron microscopy, in Figs. 7(f) and 7(i), and found that the skyrmion lattice orientation fluctuates in time among well-defined orientations. This fluctuation can provoke the observation of a Moiré pattern of skyrmions at specific locations in the sample and at specific times, see Fig. 7(g). This is due to the fact that two or more orientations can coexist during the same acquisition. As a consequence, the Fourier Transform (FT) of the corresponding image [Fig. 7(g) inset] shows the same magnetic Bragg peaks splitting as reported in Ref. 49. These results highlight that the nature of the observed splitting in the reciprocal-space time-averaged experiment is not the existence of a superposition of two skyrmion lattices originating from the two inequivalent Cu sites of material unit cell. Rather, the impact of disorder and dislocations on the skyrmion lattice orientation is found to be responsible for this observation. Our results were later confirmed by new X-ray scattering measurements using a faster acquisition time.^50^

In these experiments, the fastest time-resolution achieved was in the order of microseconds. To go beyond this limitation and completely resolve the switching between different magnetic arrangements or even coherent phenomena such as magnons,^51^ pump-probe experiments in nano- and femtosecond Lorentz TEM will be carried out in our laboratory.

## V. DYNAMICAL IMAGING OF IONS, FS SMALL ANGLE ELECTRON DIFFRACTION, AND X-RAY DIFFRACTIVE IMAGING

To retrieve the dynamical ordering/disordering properties of a self-assembled supracrystal of coated gold NPs, we applied our recently developed method of fs-small angle electron diffraction.^7^ Compared to other existing time-resolved electron diffraction machines worldwide, our instruments have a very high flux, 10^9^ e/s; this is achieved, thanks to a higher repetition-rate of the laser system (20 kHz) combined with the radiofrequency compression that allows us to store up to 10^5^ electrons in 300 fs bunches.^14^

Thanks to this apparatus, we developed an experimental scheme for small-angle electron diffraction with the aim of performing a time-resolved diffractive imaging study of different systems, ranging from nano-structures to charge ordered as well as magnetic materials. Such experiments enable recording fs and pm-resolved movies of materials and molecules with sensitivity to light elements and very thin films.

In Ref. 7, we show that in a supracrystal formed of gold NP coated by 12-atoms thiol chains, these ligands can have a preferential order. Furthermore, we also demonstrate that light excitation can serve as an external control parameter inducing further self-organization among the dodecanthiol ligands.

**FIG. 8.**
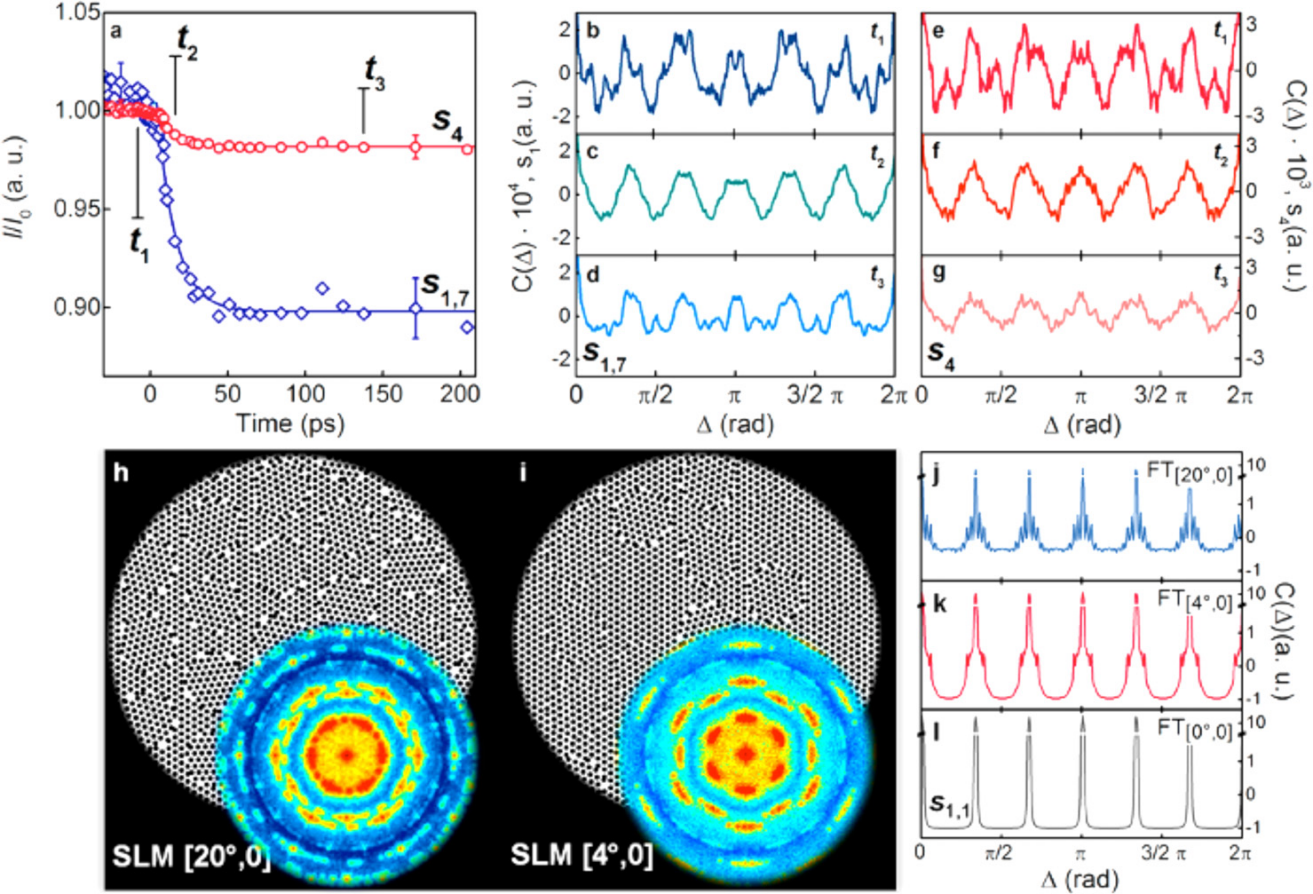
Ultrafast small-angle electron diffraction of gold NPs. Transient evolution of the intensity of the CCF function at the scattering vectors corresponding to the NP-NP distance and ligand-ligand distance, respectively. (b)–(g) CCF function at different times and different scattering vectors. (h) and (i) Simulation of disorder in the supracrystal. (j)–(l) Corresponding CCF function. Reproduced with permission from Mancini *et al.*, Nano Lett. **16**(4), 2705–2713 (2016). Copyright 2016 American Chemical Society.

In Fig. 8(a), we show the temporal evolution of the intensity of the cross-correlation function (see below) at two different scattering vectors associated with the NP to NP distance in the supracrystal and to the ligand to ligand distance in the external shell of the NPs. Because both the NPs and the ligands form glassy aggregates, we analyzed the Cross Correlation Function (CCF), which was introduced for X-ray and electron scattering in Refs. 9 and 10, respectively. In brief, such a quantity is given by the formula

$$C_{s}(\Delta )=\frac{{〈I(s,\phi )I(s,\phi +\Delta )〉}_{\phi }-{〈I(s,\phi )〉}_{\phi }^{2}}{{〈I(s,\phi )〉}_{\phi }^{2}},$$

where *I(s,ϕ)* is the diffraction intensity at a given scattering vector *s* and radial angle *ϕ*. Δ is another radial angle, as shown in Fig. 3(c).

Such a quantity has been shown to deliver information on the local symmetry of a glassy aggregate in X-ray and electron scattering experiments.^9,10^ In Figs. 8(b)–8(d) and 8(e)–8(g), the CCF corresponding to the hexagonal arrangement of the NPs in the supracrystal and the CCF representative of the ligands local order are depicted at different time-delay from the photoexcitation. While the overall intensity of the diffraction signature at both distances is found to diminish [Fig. 8(a)], as expected for a light-induced disordering effect, the shape of the CCF function is found to become better defined, with an increased visibility of its periodic modulation. This was interpreted as a transient annealing process taking place in the film due to the interplay between disordering forces and local ordering forces originating from strain in the supracrystal. A simulation of such a behavior is shown in Figs. 8(h)–8(l).

These results show the complementarity between electron and X-ray techniques. In Ref. 10, similar investigation of nanoparticles was carried out by X-ray diffraction. Several of these aggregates are interesting for application in bio-physics where the interaction between the ligands and the environment, for example a liquid, is the most important aspect. For this purpose, a probe that can penetrate longer distances in materials, such as X-rays, is more powerful. Furthermore, for larger size NPs, the larger transverse coherence of photon beams is also an advantage. In a vacuum environment and dealing with very tiny amounts of materials, electron diffraction offers unique advantages in terms of combined sensitivity and resolution, both in time and space.

## VI. DYNAMICAL IMAGING OF IONS, FEMTOSECOND PHOTOELECTRON DIFFRACTION

### A. Coherent excitation of phonons in Bi(111): Measurement of absolute delays between electronic and structural modulations

Bismuth is a well-known model system for investigating the interplay between collective modes and the electronic structure. Absorption of infrared light leads to a large displacement of electronic charge. Due to the new charge order, the atomic equilibrium positions change. In a classical picture, this situation corresponds to a strained spring, which is released leading to oscillations around the (new) equilibrium position. The model was termed Displacive Excitation of Coherent Phonons (DECP).

In bismuth, the material response to such an excitation is dominated by the A1g mode, an optical mode at about 2.8 THz polarized along the [111] direction.^52^

The DECP model was confirmed in numerous experiments (see, e.g., Refs. 53–56). Moreover, in a recent time-resolved photoemission study, it was shown that the electronic bulk states exhibit modulations in energy and intensity with the frequency of the phonon mode.^57^ However, these modulations only indirectly reflect the atomic motion. In order show that the motion can indeed be observed in the angular photoelectron distribution, we carried out time-resolved infrared pump—extreme ultra violet (XUV) probe measurements from a Bi(111) crystal. Here we take advantage of the fact that diffraction effects affect photoelectron angular distributions at both x-ray and XUV (typ. 20–100 eV) energies and can thus be recorded using high-harmonics in a laboratory-based experiment.^58–60^

Like in time-resolved x-ray absorption spectroscopy, in which both electronic and atomic structure dynamics can be observed simultaneously,^61^ the photoemission energy and angular distributions reflect both electronic structure through the photoemission density of states in the detection direction and the atomic structure by modulating the angular intensity distribution due to diffraction.

**FIG. 9.**
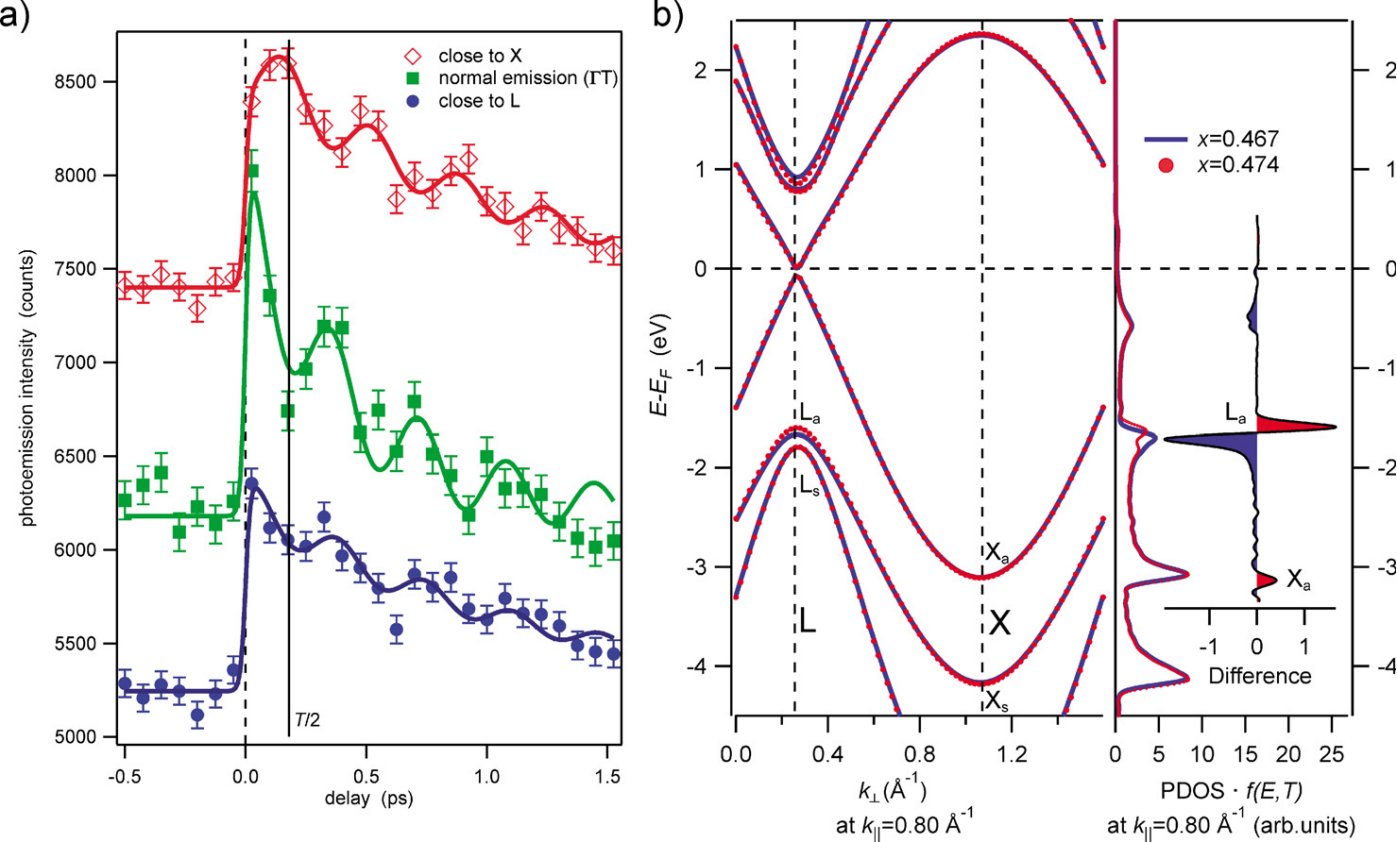
(a) Photoemission signal integrated in an energy window of 5 eV around the Fermi energy E_F_ as function of time delay for selected points in the bulk Brillouin zone: L, Γ, and X. (b) Band dispersion and photoemission density of states (right panel) for the L- and the X-point. Reproduced with permission from Greif *et al.*, Phys. Rev. B **94**, 054309 **(**2016). Copyright 2016 American Physical Society.

In Fig. 9, we show the valence band photoemission intensity recorded in a 5 eV energy window around the Fermi energy for selected points in reciprocal space.

The modulations by the 2.8 THz A_1g_ mode are clearly observed for the three transients on top of a sharp increase at time delay zero followed by a slow (∼1 ps) exponential decay. The phase, however, changes between the L- and the X-point: At L, the intensity is maximum at the onset of the oscillation and minimum after half a period *T*/2 of the phonon. In contrast to this, the signal at X shows as a weak onset only, followed by a maximum at roughly *T*/2. In the band structure, shown in Fig. 9, one sees that the conduction band minimum is located at L, while a large energy gap is found at X around the Fermi energy. The modulations at X cannot be caused by modulations of the electronic density of states but rather by changes in the diffraction pattern due to scattering at oscillating atoms.^62^

**FIG. 10.**
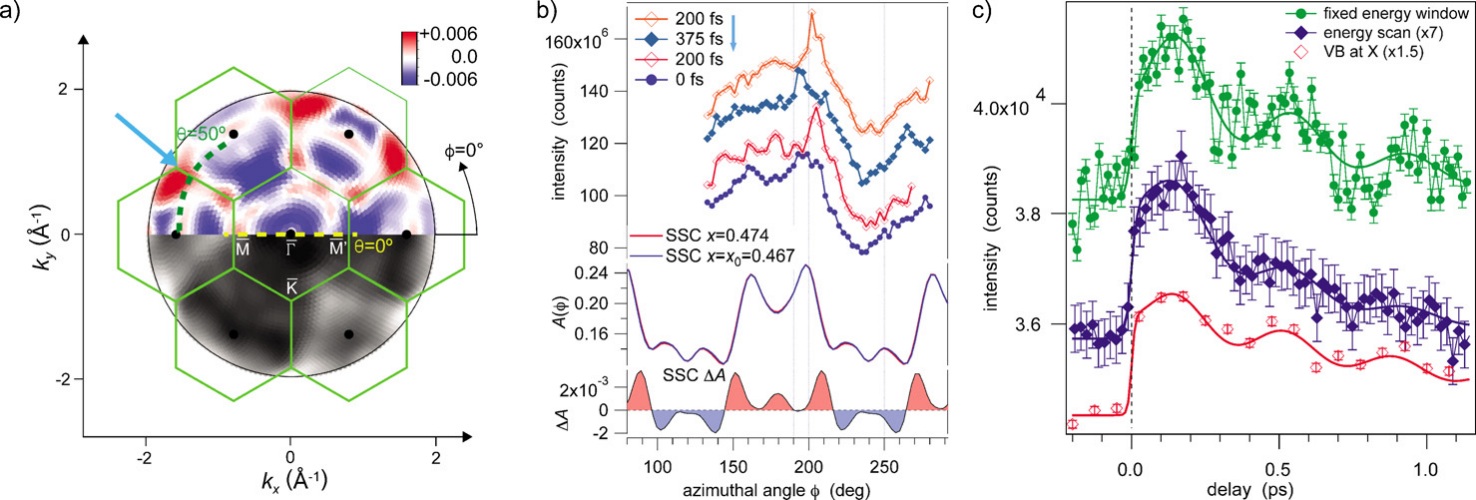
Photoelectron diffraction data from Bi(111) after coherent excitation of the A_1g_ mode by infrared light. (a) Scattering calculation showing the diffraction pattern for electrons from the 6p valence levels close to the Fermi energy (bottom half) and difference of the patterns for maximum and minimum distortion (top half): the color code indicates an increase in intensity with increasing distortion in red, a decrease in blue. The dashed green circle highlights the location of the azimuthal scan in (b), and the blue arrow highlights the location of the measurement shown in (c). (b) Intensity as function of azimuthal angle along the curve shown in (a) for various pump-probe delays. A clear change in intensity distribution is seen at about 200° as function of delay. (c) Transients recorded at the location of the blue arrow in (a) and compared to the integrated signal at the X-point.

This was confirmed by photoelectron diffraction as shown in Fig. 10: Diffraction data were obtained by integrating over the full valence band and recording the photoemission yield as a function of emission angle. Using single scattering cluster calculations for Bi in equilibrium and in the distorted state, the emission angles of maximum diffraction intensity change can be identified [see arrow and dashed line Fig. 10(a)]. Measurements along the line indicated in Fig. 10(a) are displayed in Fig. 10(b) for various time delays between IR pump and XUV probe pulse. The intensity modulation changes with delay. Time dependence at a particular angle is shown in Fig. 10(c). It exhibits the same phase as the valence band signal at the X point and directly maps the position of the Bi atoms along the [111] body diagonal.

The fact that electronic and lattice modulation are both measured directly in a single experiment allows the relative timing and phases to be determined with high precision.

The value of about 2.85 rad obtained in the present study for the phonon phase lag with respect to the electronic excitation at delay zero and the electronic modulation is significantly off the theoretical value π. This indicates that the early dynamics of the hot electron gas, which initially drive the collective phonon excitation, determine the phase lag. It is likely that the strongly excited electron gas during thermalization leads to an initial acceleration of the ions, which is much larger than the one expected once the thermal equilibrium is reached within the electron gas.^62^

We may conjecture that this kind of experiment will be able to study correlated atomic and electronic dynamics after selective excitation of particular modes in condensed matter systems.

### B. Conformational changes in molecules: Tetra-tert-butyl-azobenzene/Au(111)

Tetra-tert-butyl-azobenzene (TBA) is a derivative of azobenzene, a small molecule consisting of two phenyl moieties connected by a so-called azo-bridge, i.e., an N=N double bond. The two phenyl moieties can rotate around the azo bridge with two stable configurations, a *trans* and a *cis* isomer. Exposure to light allows the conformation to be reversibly switched between the two isomers.^63^

Adsorbed on a metallic surface, the switching ability is lost due to the presence of short-lived excited states in the substrate, which efficiently dissipates energy before the isomerization is finished. The switching ability can be restored by functionalizing the azobenzene molecules with four tert-butyl groups acting as spacers and, thereby, reducing the interactions with the metal.^64^ The detection of the conformational changes by methods other than scanning probe microscopy^65^ is difficult if the number of switched molecules corresponds to a dilute sample. In the present case, it was shown that the energy of the lowest unoccupied molecular orbital (LUMO) shifts in energy, which can be probed conveniently by two-photon photoemission (2PPE):^66,67^ the relative intensities of the two peaks corresponding to the LUMO of the *trans* and *cis* isomers equal the fraction of molecules in the respective states.^67^

**FIG. 11.**
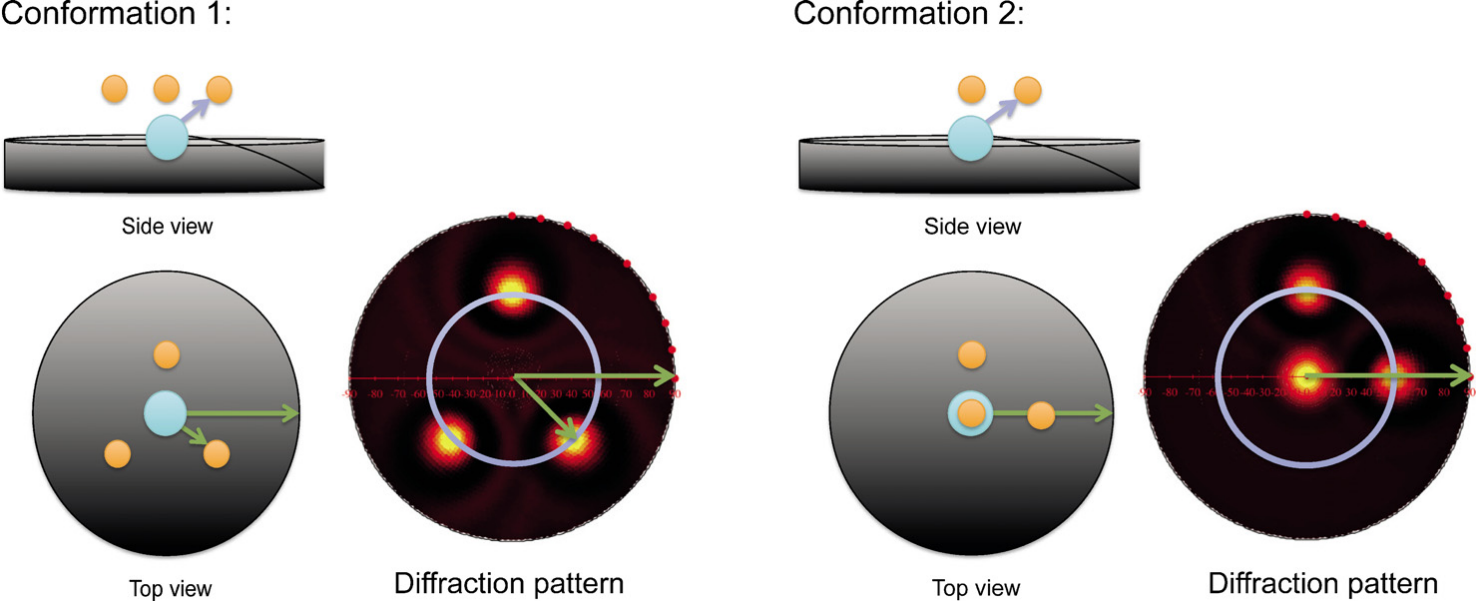
Detection of molecular conformation by photoelectron diffraction. Single-scattering cluster calculations were carried out on a molecule consisting of one emitter (blue) and three scatterers (yellow). The resulting patterns are shown on the right-hand side of the corresponding molecular structures. A switch of the molecule is mimicked by the displacement of two scatterers into new positions relative to the emitter. The scattering pattern changes accordingly which allows the two patterns to be used as fingerprints for the two isomers.

In order to access the fraction of switched molecules directly by a structural probe and to estimate switching rates, we employed x-ray photoelectron diffraction from TBA/Au(111) under different illumination conditions.^68^ The diffraction patterns of the two isomers are used as fingerprints as shown schematically in Fig. 11, and their relative contributions to the pattern equal the ratio of the respective concentrations in the sample.

**FIG. 12.**
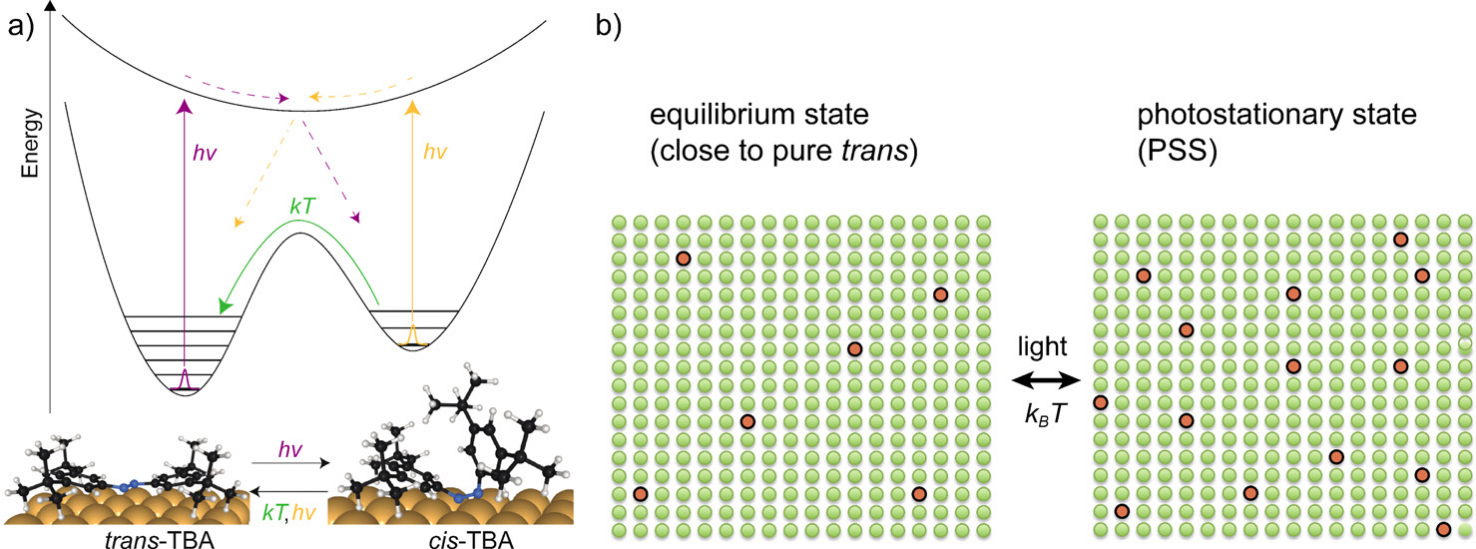
Reversible isomerization of TBA molecules on Ag(111). While the net thermal switching rate drives the sample towards a pure trans state at room temperature, absorption of light leads to isomerization in both directions with a higher cross section for trans-to-cis isomerization.^67^ The two states are sketched qualitatively on the right-hand-side: the photostationary state corresponds to a sample with increasing concentration of cis isomers (red circles).

The principle of the experiment is depicted in Fig. 12: the room temperature equilibrium state is close to a pure sample with *trans* isomers only.^67^ Upon illumination by uv-light, some of the molecules undergo reversible switching. The switching may proceed in either direction with a higher probability for a *trans* to *cis* isomerization.^67,69^ This means that the dynamical equilibrium is shifted towards higher *cis* concentration. We label the new equilibrium state photostationary state (PSS).

**FIG. 13.**
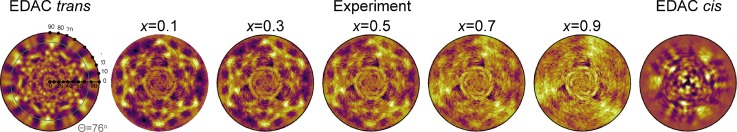
Experimental patterns obtained by subtracting the pattern of the pure sample, scaled by a factor x, from the pattern of the sample exposed to uv-light. Reproduced with permission from Schuler *et al.*, Struct. Dyn. **4**, 015101 (2017). Copyright 2017 AIP Publishing LLC.

Since the molecules are independent even for monolayer coverage, the PSS represents a mixture with a concentration of *cis* isomers, which is higher than the one in the equilibrium state. Two XPD patterns were recorded, one for the equilibrium at 300 K and the second one with the sample exposed to a high-intensity uv lamp (PSS). In order to quantify the ratio of *trans* and *cis* isomers in the PSS, the equilibrium pattern was scaled with a factor *x*, 0 < *x *<* *1, and subtracted from the pattern of the PSS. The result was compared to multiple-scattering Electron Diffraction in Atomic Clusters (EDAC) calculations^70^ based on electronic and structural calculations for TBA/Au(111).^71^ Some patterns for selected values of *x* are shown in Fig. 13.

One can easily see that the difference pattern approaches the EDAC calculation of the *cis* state. A more quantitative assessment allows us to estimate the fraction of switched molecules to about 8% under these conditions and at 300 K.^68^ This highlights the fact that conformational changes can reliably be observed even for dilute samples with only orientational order.

## VII. CONCLUSIONS

Time-resolved techniques based on the detection of electrons are a valuable complement to X-ray scattering methods because they allow access to different length-scales (in the atomic to nm regime) and a good sensitivity to light elements and tiny amounts of material. Combining different methods based on imaging, diffraction, and spectroscopy, we have shown that details on atomic motions, charge, and spin dynamics can be obtained in nanostructures and surfaces.
